# Greening the gentrification process: Insights and engagements from practitioners

**DOI:** 10.1177/25148486241236281

**Published:** 2024-03-07

**Authors:** Jessica Quinton, Lorien Nesbitt, Daniel Sax, Leila Harris

**Affiliations:** Department of Forest Resources Management, 8166 The University of British Columbia, Vancouver, BC, Canada; Institute for Resources, Environment and Sustainability, 8166 The University of British Columbia, Vancouver, BC, Canada

**Keywords:** Urban forestry, Canada, environmental gentrification, environmental justice, green space

## Abstract

Green gentrification implicates urban greening as a driver of neighbourhood ‘upgrading’ and subsequent displacement. However, it is unclear whether the concept resonates with, or supports the work of, those responsible for much of the greening occurring in cities – urban green planners/practitioners. We interviewed 33 planners/practitioners in Canada to refine our understanding of the relationships between urban greening and gentrification. We found that greening is closely tied to development, with funding/space for greening often provided through development requirements/incentives. Thus, rather than greening *causing* gentrification (as described in current literature), here greening is often a *requirement* and *direct outcome* of new development – contributing to what we describe as a broader *greening of the gentrification process* that is facilitated by various political-economic factors. Many interviewees stated that their current work focuses on addressing existing inequities rather than strategizing to limit future gentrification. However, they had mixed opinions about whether knowledge of green gentrification as a concept can help them promote equitable urban greening due to their lack of power over where/how urban greening occurs, along with the finding that greening is not causing gentrification. The uneven power dynamics between urban green practitioners/planners, developers, and elected officials also influenced views on whether gentrification is an intended outcome of greening. We conclude that relying on new development to provide urban greening is antithetical to addressing existing green inequities and is likely to exacerbate inequities through associating greening with gentrification. Recent measures to improve housing affordability (i.e. the removal of developer greening requirements) will disrupt the current development-greening relationship but are unlikely to address the issue of inequitable greening. Increased and ongoing collaboration between those working in urban greening, housing, and planning is paramount and should focus on affordability and equity across urban systems – attending to the interplay between greening, housing, affordability, and sustainability.

## Introduction

Cities are increasingly recognizing and attempting to address (Grant et al., 2022; Nesbitt et al., 2019) existing inequitable distributions of urban vegetation (Gerrish and Watkins, 2018; Quinton et al., 2022; Rigolon, 2016). However, concern has recently arisen about green gentrification, wherein greening initiatives (e.g. parks, community gardens, etc.) result in higher-income households moving in and displacing existing residents (Checker, 2011; Dooling, 2009; Gould and Lewis, 2017), which upholds existing, and creates future, green inequities. Numerous quantitative analyses have found an increase in household incomes, property values, education levels, etc., surrounding new greening interventions – although such results vary depending on proximity to downtowns (Anguelovski et al., 2017; Rigolon and Németh, 2020), types of green spaces (Triguero-Mas et al., 2022), and their size (Chen, 2021; Kim and Wu, 2021; Rigolon and Németh, 2020). Case studies of the NYC High Line have demonstrated how increased property values are used to rationalize/motivate greening projects (Lang and Rothenberg, 2017; Loughran, 2014; Patrick, 2014), and the Atlanta Beltline showed how simply announcing such greening projects can result in property speculation surrounding them (Immergluck, 2009). Beyond physical displacement, green gentrification can negatively impact long-term residents, such as increased policing (Harris et al., 2020), exclusion from new green spaces (Patrick, 2014; Pearsall and Eller, 2020), and environmental decision-making (Checker, 2011; Miller, 2016), and a reduced sense of belonging/community (Goossens et al., 2019; Jelks et al., 2021).

Some have framed green gentrification as an unintended consequence of addressing existing green inequities (Pearsall, 2018) while others suggest it is an intentional process that uses greening to disguise negative social impacts (Dooling, 2009), attract high-income households (Gould and Lewis, 2017), and/or co-opt environmental-justice narratives to serve uneven economic growth (Checker, 2011). The latter highlight green gentrification as a ‘sustainability fix’ (While et al., 2004) and/or an example of ‘greening the growth machine’ (Dilworth and Stokes, 2013; Molotch, 1976), wherein urban greening efforts are selectively incorporated into policy/planning agendas to increase urban land values to generate uneven economic growth. The placing of green (or environmental or ecological) alongside ‘gentrification’ is used by some researchers (e.g. Anguelovski et al., 2019; Dooling, 2009) to re-politicize urban greening and its consequences (whether intentional or unintentional), as greening is often portrayed as apolitical and good for everyone (Angelo, 2020).

Recently, a process of ‘gentrified greening’ has been identified, in which greening follows rather than precedes gentrification (Sharifi et al., 2021) due to the leveraging of developer funding and gentrifier resources to green gentrified neighbourhoods (Rigolon and Collins, 2022). Processes of green gentrification and gentrified greening can be difficult to disentangle, and it has been posited that in some instances they may occur within the same area as part of a ‘green gentrification cycle’ (Rigolon and Collins, 2022). The green-gentrification cycle highlights just some of the complexity of understanding the relationship(s) between urban greening and gentrification. Much research on greening and gentrification has focused on case studies and quantitative analyses of select greening types/interventions (and often, spectacular interventions like the NYC High Line). However, there is a need to consider the broader political-economic and social contexts/processes underpinning the relationship(s) between urban greening and gentrification (Sax et al., 2022b). In the following subsection, we outline some aspects of these broader contexts related to urban sustainability, planning, and development – and thus, related to green gentrification. The rest of our paper draws primarily on interviews with urban greening planners and practitioners to examine how these contexts/processes influence the relationship between urban greening and gentrification in the Canadian regions of Metro Vancouver and the Greater Toronto Area.

### Urban planning and development in an era of neoliberal sustainability

Sustainability (and related concepts like livability and resilience) have become central to urban planning/policy agendas over the past several decades (Gunder and Hillier, 2009; Wheeler, 2013). Hegemonic approaches to urban sustainability have been criticized for being neo-managerial (i.e. focused on indicators, rankings, and awards developed by public and quasi-public authorities), post-democratic, oriented towards uneven economic growth, and based upon uncritically replicating ‘best practices’ (Rosol et al., 2017). Current growth-oriented approaches to sustainability are unsurprising given that the sustainability shift has occurred within a broader political-economic context of de-industrializing urban cores (MacKinnon and High, 2020), the move from urban managerialism to entrepreneurialism (Harvey, 1989), a decline in social spending and support (Harvey, 2005), and the financialization of housing (Aalbers, 2016).

Canadian cities are no exception to these trends and have increasingly emphasized urban sustainability planning since the early 1990s (Keil and Graham, 1998). Many have set ambitious goals regarding tree canopy cover, park accessibility, local food production, and so on (as seen in e.g. Vancouver's Greenest City 2020 *Action Plan* and *VanPlay: Vancouver's Parks and Recreation Master Plan*; Toronto's *Strategic Forest Management Plan* and *GrowTO Urban Agriculture Action Plan*). Vancouver in particular has endeavoured to establish itself as the ‘greenest city’ (Affolderbach and Schulz, 2017). In response to concerns about (and in some cases, physical limits to) suburban sprawl, cities have incentivized densification by allowing increased development heights and densities in exchange for various community amenity contributions, including greening contributions like new or improved parks, streetscapes, and urban forests (Moore, 2016). At the same time, provincial involvement in – and funding of – urban affairs was reduced (Taylor, 2019), and municipalities have increasingly funded large development projects via public-private partnerships (Vining and Boardman, 2008). Housing prices and household indebtedness have soared in many Canadian cities (Daoust et al., 2021; Statistics Canada, 2022), particularly in this study's focal regions of Metro Vancouver and the Greater Toronto Area (Gordon, 2020). In Vancouver, these prices are very detached from local incomes (Gordon, 2020). These regions have also been experiencing gentrification over the past several decades – particularly in downtowns and along transit corridors (Grube-Cavers and Patterson, 2015; Walks et al., 2021).

The political-economic and sustainability contexts described here underscore the potential for urban greening in Canadian cities to become implicated in gentrification processes. Limited green gentrification research has been conducted in Canada (Quinton et al., 2022), but suggests variable and complex relationships between greening and gentrification within and between cities (Anguelovski et al., 2022; Bunce, 2018; Parish, 2020; Quastel, 2009; Sax et al., 2022a; Triguero-Mas et al., 2022). This study aims to generate further insight into the relationship(s) between greening and gentrification in Canadian cities (which extend beyond the current emphasis on greening *causing* gentrification) by exploring how urban greening practitioners and planners understand, experience, and perceive the concept of green gentrification.

### Urban greening planners and practitioners and green gentrification

Some green gentrification research has engaged with urban greening practitioners and/or professionals in allied policy/planning fields to understand the process in specific case studies (Quinton et al., 2022), but there has been little research on their perspectives about, and use of, the term/concept itself or how practitioners/planners might work proactively to mitigate negative impacts of green gentrification (but see Rigolon et al., 2020). Due to their influence on the greening of public (and to some extent, private) lands (Konijnendijk et al., 2006; Low et al., 2005), these practitioners can provide key insights into how urban greening occurs within the broader political-economic context and the implications this has for green equity and gentrification. Such information is critical for mobilizing research to practice and refining existing theories on the relationships between urban greening and gentrification.

This study builds on our previous survey (Nesbitt et al., 2023), which found that greening practitioners have heard the term ‘green gentrification’, and associated it with physical displacement, but rarely incorporate it into their work. To gain a nuanced understanding of their insights/perspectives on green gentrification, we interviewed greening practitioners/planners in Metro Vancouver and the Greater Toronto Area (GTA). We then presented the themes constructed based on the interview data to focus groups, allowing participants to confirm or amend our interpretations. Both regions engage in urban green boosterism, leveraging sustainability branding to garner global attention (Garcia-Lamarca et al., 2019; McCann, 2013). As mentioned previously, they also show dramatic decoupling of household incomes from local real-estate markets (Gordon, 2020) and have experienced ongoing gentrification (Walks et al., 2021), making them interesting focal points for this research. Our objectives were to understand planner/practitioner perspectives on:

1. Connections between their work and gentrification.
2. How urban greening becomes connected to gentrification in their work.
3. Potential methods of mitigating green gentrification.
4. The relevance and usefulness of the term/concept of green gentrification for promoting green equity within their work.

Our study draws on empirical data to expand upon existing conceptualizations of the relationships between greening and gentrification (i.e. that greening *causes* gentrification or vice versa) by highlighting how urban greening requirements/incentives levied on new developments result in a broader *greening of the gentrification process* reflective of the sustainability shift in urban planning/policy agendas. This broader greening of gentrification results from the co-location of urban greening and development targeted towards higher-income households, which results in greening being a common feature of gentrification – but not as a driver of the process. We highlight nuance in the intentionality of green gentrification and relate this to the uneven power dynamics between developers, politicians, and those responsible for urban greening and planning. These power dynamics also influence interviewee perspectives on the usefulness of the concept of green gentrification for creating more equitable green cities. We also describe emerging concerns about the impact of recent housing affordability measures on urban greening, which have the potential to influence future dynamics between greening and gentrification.

## Methods

**Figure 1.**
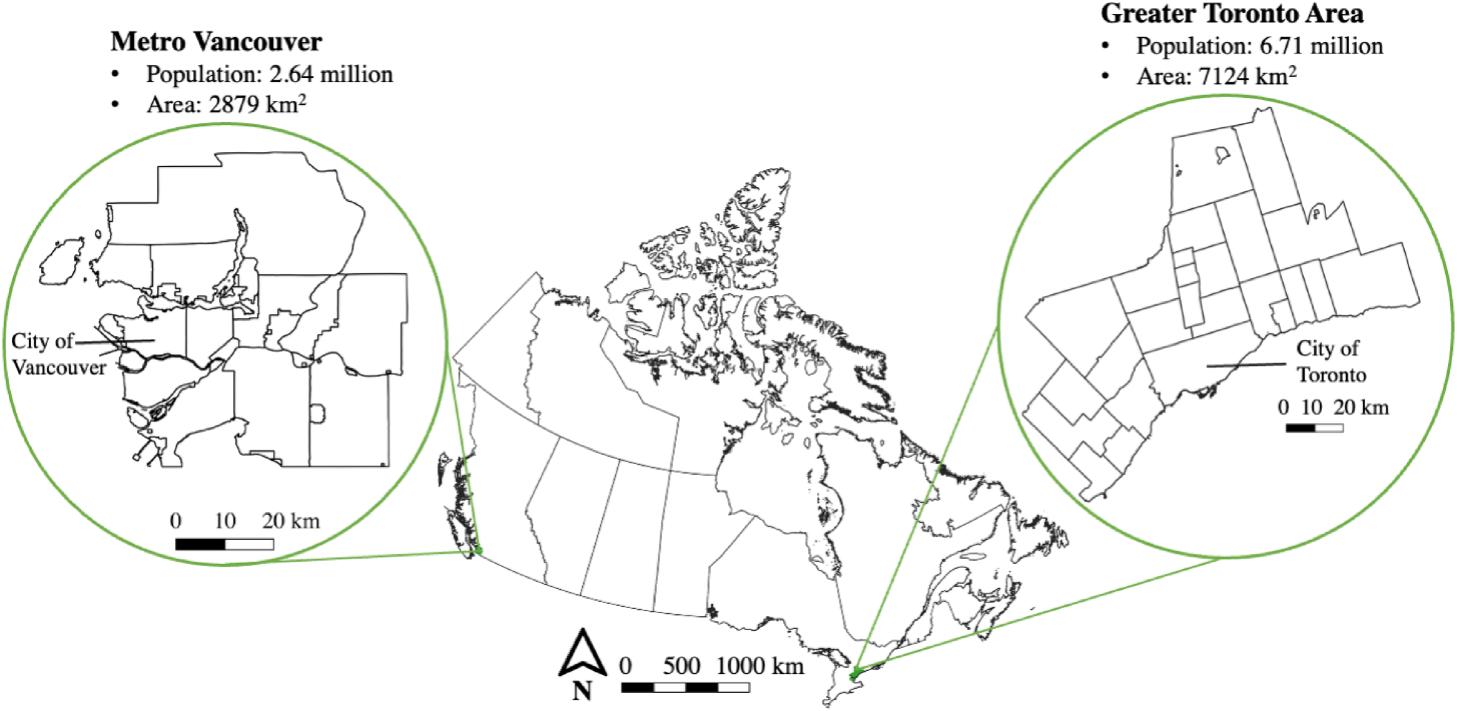
Metro Vancouver and the Greater Toronto Area, Canada (boundary files from Statistics Canada, 2021).

In February/March 2023, we conducted semi-structured interviews with 33 individuals working in urban planning and/or urban greening (e.g. urban forestry, parks, green infrastructure, etc.) in the regions of Metro Vancouver and the GTA (Figure 1). Both regions include a major city (Vancouver and Toronto, respectively) surrounded by suburban and exurban municipalities of varying sizes and densities. Local governments have primary authority over urban planning/policy, but these matters are also influenced by regional and provincial governments (Taylor, 2019).

Participants were recruited by (i) following up with participants from the survey (Nesbitt et al., 2023); (ii) snowball sampling based on their suggestions; and (iii) purposive sampling targeting underrepresented areas of work. Most participants were municipal/regional government employees (*n* = 24; many from the major cities of Vancouver and Toronto), but some worked for consulting firms (4), environmental NGOs (2), or conservation authorities (3) that work across multiple cities. They had varying positions and years of experience. Their work included planning, policy, design, management, and/or maintenance of parks (8), urban forests (4), green infrastructure (2), natural areas (2), or some combination of these (13). Some interviewees worked in general urban planning/policy (4). Some participants had worked in more than one position or city in the metropolitan area and were able to draw on insights from various positions/organizations.

Participants were asked whether their work contributes to gentrification generally; their familiarity with and understanding of the concept of green gentrification; if they could identify areas of previous, current, or probable future green gentrification; what they can – and cannot – do to limit negative gentrification outcomes resulting from greening; whether knowledge of the term/concept of green gentrification is useful to promote green equity within their work; and what concerns they have with the concept. All interviews took place over the Zoom video-conferencing platform, were audio-recorded, and later transcribed verbatim using Otter.ai.

### Analysis

Interview transcripts were analysed in NVivo (QSR International Pty Ltd, 2020). Transcripts were sorted into organizational categories (Bingham and Witkowsky, 2022), such as metropolitan region, greening type discussed, etc., to add further nuance to the analysis. A multi-cycle and iterative coding approach was employed. Initial coding took an in vivo approach to develop codes based upon participant language (Saldaña, 2016). This was followed by thematic analysis (Braun and Clarke, 2006) to identify broader patterns in the data. Themes were presented to participants in four focus groups held in July 2023, and they were asked to reflect on the findings individually and in pairs before providing feedback to the researchers to ensure the themes reflected their perspectives and experiences.

The analysis took a reflexive approach to ground interpretation in the context and reality of participants and acknowledges the centrality of the researchers and their positionality in the process of making meaning from the data (Richards, 2022). This research is informed by a constructivist worldview in which meaning is subjectively created by individuals and influenced by their context (Creswell, 2006). The research team is mainly composed of scholars whose educational and research backgrounds are focused on urban greening and environmental justice. Some participants were known to the research team through previous interactions and first heard about green gentrification from them.

## Results

**Figure 2.**
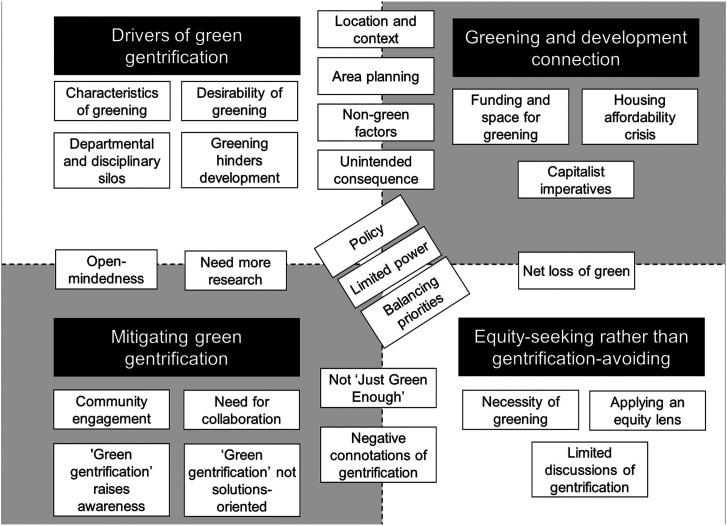
The four major themes (black boxes) and sub-themes/codes (white boxes) were identified through interviews.

Four major themes (Figure 2) were identified from the interviews: (i) what (and who) is driving gentrification; (ii) the close connection between urban greening and development; (iii) a clear emphasis on addressing existing inequities rather than limiting future gentrification; and (iv) the need for collaboration, policy, and community engagement to mitigate negative outcomes of green gentrification – primarily, physical displacement. Numerous sub-themes were identified, many of which fell under more than one major theme, highlighting their interconnection. Notable overlapping sub-themes included the need for policy and the limited power felt by interviewees. These themes and subthemes were affirmed by focus-group participants.

### What (and who) is driving green gentrification?

#### Desirability and characteristics of greening

When asked to define green gentrification, most respondents linked greening to gentrification by describing greener neighbourhoods as more desirable places to live, which increases housing costs and attracts higher-income households to move in:

I think people who have the means to choose where they want to live, I think a lot of them value living near green space. And I think those areas are now more than ever seen as high-value areas. (GTA_1)

I've been doing this kind of work for years now. And I do know that when we put a shiny new park into a neighborhood, or our neighborhoods that have a higher density of parkland, they tend to have higher property values. (MV_15)

However, they did not view all greening as having the same gentrifying impact. Parks, particularly large parks, were most readily linked to gentrification. It was harder for interviewees, particularly those working primarily with urban forests or green infrastructure, to see the link with greening spread throughout the city, such as street trees and small green infrastructure:

So I can see how a major investment in greening on the scale of the Highline […] could drive gentrification, but I think activities like street-tree planting and things like that are probably not really going to tip the scales. (MV_3)

#### Non-green factors driving gentrification

Interviewees frequently expressed a lack of conviction that greening was actually driving gentrification, and rarely believed it to be the only contributing factor. They noted that greening is often occurring alongside other non-green initiatives such as new transit, housing, community centres, and so on.

So the park aspect is part of it, but it's the whole kind of amenities-service-delivery-connectivity-transportation stuff as well. (GTA_12)

…the green tends to be part of something larger. Our transit corridors, so yeah, we greened our transit corridors but *we built transit corridors*. And that's what's changed the character of those neighborhoods—not that we greened them. (GTA_7)

Some noted that greening requirements for new developments (e.g. retaining existing or planting new trees, creating new parkland) are more of a nuisance to developers due to the extra costs and reduction in the total developable area of the site. This suggested to some interviewees that developers do not view greening as a gentrification tool to generate greater profits.

Does the work that we do contribute to gentrification? No, we are more of a hindrance […] Developers and builders etc., want to capitalize on as much space and land that they have available to them, which is very often at the expense of the natural heritage system – parkland. (GTA_16)

They were, however, open to changing their opinion on the link between greening and gentrification if presented with additional research/data. Many indicated a desire for more research, particularly local research, to tease out how much influence greening has relative to other factors:

I would love to see the research […] that has tried to pick apart what is driving gentrification in the neighborhood. And how much of that is related to greenery versus something else? (GTA_3)

### Departmental and disciplinary silos

Many interviewees noted that their work focuses exclusively on urban greening or environmental work with limited scope to consider other factors such as housing and the potential impact of greening on them. Several people described an antagonistic and adversarial approach to area planning, where they feel their job is to advocate for as much greening as possible, in opposition to other budgetary or infrastructure priorities. Their hope was that employees in other departments were advocating for affordable housing, social policy, etc., to balance out their focus on greening. Those working as more generalist planners, and some working in greening, were conscious of the problems with this approach:

So it's the single-issue advocacy, I find is consistently problematic. And it does tend to happen more with people that are advocating for ecology or green space – they seem the most consistently unaware or forgetful about the topic of gentrification and displacement. (MV_2)

Similarly, those working in environmental NGOs noted that their funding is tied to greening initiatives, meaning they cannot undertake other non-greening work. One participant linked this to an ‘environmental saviourism’ attitude, in which it is believed that greening is always a positive action benefiting everyone.

I think that environmental saviourism attitude is very much alive and well, and most folks in this organization have good intentions without realizing that environmental greening interventions are probably not always the only solutions. But because of the way we're funded, the way our organizations are designed, and the way our priorities are set, that's what we're pushing for. (GTA_17)

People felt they had limited ability to influence where greening occurs or its connection to gentrification due to the scope of their work and their position within their organization. It was frequently described as an “unintended consequence” of their work, and this view appeared to be due to (i) the lack of power they had in greening actions (e.g. choosing where to green) and over housing and social policy; and (ii) a belief that greening is always good. It was also evident that interviewees were not evaluating the social impacts of their work nor considering potential negative outcomes.

[A]s an urban forester, or somebody who plants trees, I can only plant where there's space to plant trees… Really, I’m working with what I’m given. (GTA_2)

I don't think that by any means does our work intentionally promote gentrification. (MV_11)

I can't even begin to quantify the positive or negative impacts of my work. It's just the work that I do. And I hope it's good. It's parkland, it's got to be good. But as you know, there are unintended consequences, and we do not engage with them at all. (GTA_13)

### The greening-development connection

It was evident, particularly from participants in the GTA that much of the funding and space for greening comes through development (e.g. through parkland provision requirements, community amenity contributions, density bonusing, etc.). This was explained to be a result of relying on development to fund new urban infrastructure in exchange for keeping property taxes low. Some also mentioned it was a consequence of reduced provincial and federal funding for urban infrastructure.

[D]evelopment drives everything here … you generally have nicer infrastructure, whether it's green infrastructure, hard infrastructure, near new development. And I think that's because we budget in a way for things to be almost self-sufficient. We've relied on new development driving improvements, and keep property taxes low. And that's kind of the bargain that the politicians here have made. (GTA_13)

In the case of the City of Vancouver, one planner noted that those working in urban greening try to leverage new development to fund specific greening projects:

The way that ask comes in, is, let's say the engineering department is responsible, or the parks department is responsible for making the ask, they'll say, ‘hey, people responsible for land-use regulations, can you build or allow expensive strata buildings, so that we can use it as a funding source to pay for this thing?’ So it's not that the green feature is causing the gentrification, it's that the objective to add the greenness causes a change to the housing policy, or the land-use and development policy that causes a change in the income of the people that can live in an area. (MV_2)

Due to the high land costs in these regions, cities are becoming less likely to take cash-in-lieu of parkland requirements levied on new developments, which often results in new parks being located near them rather than being targeted to other areas needing parkland:

[W]hat we're finding is that we struggle with keeping up with real estate, the increased real-estate costs, so that now we're more inclined to take our land where we can get it, when we can get it as part of development. (GTA_11)

Some explicitly identified the broader capitalist system and its growth imperative as being central to green gentrification outcomes. Interviewees were acutely aware of the high housing costs in these regions, and several noted that new developments are typically targeted towards higher-income households. Although noting green gentrification as an unintended consequence of their own work, some believed it to be the intention of politicians and developers who are aiming to generate economic growth through development targeted towards wealthier residents.

The banks support the development industry by providing them loans and what have you. And if they were to exercise more controls, and have more of a green moral compass rather than a capitalistic moral compass, things might differ. (GTA_16)

I wouldn't be uncomfortable saying that probably a majority of Toronto's Council also, they maybe wouldn't use the word gentrification because it has the baggage, but they are implicitly, whatever, they might call it value uplift or something, right? Like, there's a lot of that from the council side to where they'll say, ‘I really want this area to be improved, I think this development improves this area.’ (GTA_13)

### Equity-seeking rather than gentrification-avoiding

#### Addressing existing inequities

Many interviewees noted that their work takes an ‘equity lens’ that focuses on addressing existing inequitable distributions of urban vegetation rather than future potential inequities created by green gentrification.

… the city is trying to do more greenery in areas that we see there is not that much, but I don't think they're doing it with the intent of resulting in gentrification. I think we're focusing on that to reduce the inequity in terms of environmental justice. (MV_13)

… we've looked at it from the other perspective of equity-seeking rather than gentrification-avoiding. (MV_4)

However, relying on new development to provide funding and space for urban greening makes it difficult to address such goals and may ultimately promote green gentrification. Although they are aware of areas that are deficient in greening, various political-economic factors (e.g. high land costs, preferences/desires of politicians, etc.) result in greening placed alongside new developments – even if these are not the areas most in need of greening

… where you struggle with our goals in trying to deliver new parkland to areas that might be low-income or are in need of more parkland but that aren't getting the development, which then could be characterized as gentrification. So there's a little bit of a Catch-22 there in how we get parkland through development, right. (GTA_11)

#### Necessity of greening and balancing priorities

Interviewees noted the necessity of urban greening and the myriad benefits that it provides to residents. In Metro Vancouver, this was often tied to the recent Coroner's Report on heat-related deaths and the recommendation that greening should be undertaken to cool cities.

It would be tough for us to make trade-offs in our recommendations, especially in an underserved area, like the Downtown Eastside. So as far as strategy, Vancouver is a good example where we're actually prioritizing greening in those neighborhoods. And, you know, the Coroner's Report is saying that's part of what needs to happen to reduce the vulnerability of those neighborhoods. (MV_3)

Some did note, however, that the public, developers, and colleagues in other departments, sometimes view urban greening as more of a luxury and believe it to be less of a priority than other infrastructure.

And a lot of people, even just the public, they say, green infrastructure or any sort of greenery, it's a ‘nice to have’, in a lot of cases. It's kind of unfortunate, but it's kind of how it is. (GTA_14)

Some noted the desire to, and challenges of, balance between urban greening to address existing inequities while minimizing the potential for green gentrification. They were aware that the neighbourhoods currently lacking in greening were typically amongst those most susceptible to gentrification, but it was not clear to them how to balance the need for greening with the need to limit gentrification.

I do think for me that the piece that gets really messy is the intersection between equity and gentrification, because the balance of benefits and disbenefits, it is a hard one to strike. (MV_4)

Others, meanwhile, were more explicit about their focus on addressing existing inequities and trying to increase urban vegetation overall due to their belief that the benefits of greening far exceed any potential negative social impacts.

I feel like trying to expand the city's canopy cover is a way more important thing to focus on than this nebulous piece of gentrification because of the overall benefits that good canopy will provide to its residents. (MV_14)

#### Limited discussions of gentrification and discomfort with the term

It was clear that there were limited discussions of green gentrification in most workplaces. The few participants who had heard the term at work noted that the discussions were brief because it was unclear whether it applied to their work or what could be done about it. Conversations around existing inequities were much more commonplace.

I don't think that green gentrification is something that's really on the PFR [Parks, Forestry & Recreation] radar, to be honest. (GTA_10)

Nobody in my team has ever brought the term up. (MV_15)

It's kind of interesting using the term gentrification … we just don't use that a lot. I've heard equity everywhere. I've literally heard it five times in the last 24 h in different contexts. So everybody's talking about equity, but I don't think they necessarily think about it as gentrification. (MV_7)

Several people noted the negative connotations of the word ‘gentrification’ primarily due to its association with displacement. When asked to define green gentrification, many interviewees described increased housing costs and associated physical displacement as an outcome. However, some respondents specified that gentrification can be positive, noting it does not always result in displacement.

… gentrification is sort of like, *gentrification …* it's displacement. (MV_9)

… I don’t mean to vilify gentrification. I think gentrification can be really good for a city too … but I don’t want that to be at the cost of pricing people out of the city. (GTA_10)

There was a discomfort with, or defensiveness against, the kind of solutions the term implies – namely, that greening efforts should be limited. This was a point reiterated in several focus groups. There was also some concern that they were being blamed for causing gentrification despite, as highlighted above, the lack of power they felt they had over gentrification and some aspects of greening. All of these factors may have contributed to the limited discussions of these terms.

… it's triggering a little bit because I'm thinking ‘does that mean, we're not supposed to, you know, protect green spaces, add green infrastructure because we're gonna push people out of the community?’ (MV_15)

I feel like it sometimes points blame at practitioners for gentrification, and the truth is, if there were no trees in Toronto, there would still be gentrification. (GTA_2)

### Mitigating negative outcomes of green gentrification

#### Using ‘green gentrification’

In addition to being concerned about the negative connotations of gentrification, several participants felt the concept is not oriented towards solutions, which makes it less useful to promote green equity through their work. In particular, some felt ‘green’ gentrification to be a misnomer, both due to their belief that it is not greening alone driving gentrification and that reducing greening would not be an effective solution for limiting gentrification and could, in fact, perpetuate existing inequities.

I feel like it doesn't tend to be solutions-oriented, that it tends to just be a way of preventing investment, but it doesn't discuss how to change that investment or how to work towards a solution. (MV_6)

… it's one thing for me to bring it to the attention of the municipality that the work we're doing with them might result in green gentrification, but if I can't bring that up to them with some kind of a solution, or at least a direction to take them, then that's really where we get challenged. (MV_4)

[…]the focus is on how do you manage gentrification and displacement first, and then the greenness is kind of a subset of that […] So it's more generally ‘how can we limit gentrification and displacement? Or have gentrification without displacement in a city?’ and what are the most effective tools for that? (MV_2)

An initial conclusion many participants drew was that ‘green gentrification’ implies that the solution is to reduce greening efforts – a ‘solution’ many were not willing to consider. A few participants explicitly mentioned (and disagreed with) the ‘Just Green Enough’ hypothesis (Curran and Hamilton, 2012, 2018), which was initially conceptualized to counter-hegemonic approaches to urban greening that favour aesthetic/spectacular greening tied to development. It aims to situate environmental remediation and amenities alongside continued industrial use and blue-collar jobs. However, it has often been interpreted as simply suggesting greening in general should be reduced to mitigate gentrification.

… there might be the implication that greening should be avoided, because it has those implications, right, of gentrification. I think that would be maybe not the right connection to make. (GTA_12)

[W]e don't want to stop planting trees in low-income areas because, you know, that's actually wrong. (GTA_7)

Those who could see ‘green gentrification’ being a useful term in their work sometimes had difficulty envisioning how exactly it could be used to support equity. Most people who thought it was useful pointed to its ability to raise awareness of the potential negative social impacts of greening.

I think green gentrification would be a tool to communicate, to help us achieve the quality of life that we want, the great access to green space, the benefits of nature, but also being cognizant of its potential negative impacts on property values and housing affordability. (GTA_3)

I think there's a lot of potential, and perhaps the use of these terms might get the attention of higher-ups. So when we bring a term like equity, and equity around neighborhoods, or green gentrification, it gets more attention. (MV_13)

#### Limited power and the need for policy

Many interviewees noted their limited power to take action on negative green gentrification outcomes, whether this was because of their position within the organization or the level of government for which they worked.

If we really want to focus on real change, it has to come from the province. So if the province isn't really going to do anything, if they're not willing to do anything, it's hard for municipalities to go out on their own and make some of these changes. (GTA_14)

… managers, directors, we can advocate and we can feel strongly about something or a particular development or what's happening, but ultimately, decision making, you know, we have very little influence, like, it's our decision-makers and the direction that we get. (MV_11)

The need for strong and enforceable municipal and provincial policy, particularly around housing affordability and provisions for urban greening during development, were often cited as necessary to mitigate physical displacement and support equitable greening. This typically fell outside the scope of their work, and many felt policymakers had a much greater ability to mitigate negative green gentrification outcomes. In discussing solutions to limit green gentrification, several interviewees reiterated their lack of power as a rationale for why they should not be blamed for green gentrification.

… it's layers back, it's higher levels of policy that designers don't touch. And I think that just pointing to the design of a park as increasing the value is too easy of a solution. (MV_6)

[…] it's the policymakers at the city that have the biggest role to play because they're the ones who are setting policies that require development to meet certain standards of rental replacement, or providing affordable housing or ensuring that lower-income people are not displaced by new development. (GTA_10)

[…] I think there are a lot of factors beyond me planting trees that are going to influence gentrification in our neighborhoods. It will be more biting, probably a lot of planning policy will have a great influence on it. So we'll see if Doug Ford gets voted in in four years. It'd probably be the biggest determining factor. (GTA_7)

#### Community engagement and collaboration

To overcome the adversarial planning process and advocating for single issues, many interviewees highlighted the need to collaborate more, particularly with housing departments and organizations:

And I know that there are some tools like some policy toolkits out there […] And I […] understand many of the tools to need interdepartmental collaboration to an extent that we don't really actually see in the city. […] So I think more interdepartmental collaboration and coordination is needed, especially with folks in social policy and housing. (MV_16)

… it used to be kind of like, ‘okay parks planners do this, and engineers do this. And social planners, you know, do this, and bylaw officers and police and fire emergency services do this and economic development…’ but more and more, we have to work as a team on almost every single project because they're just complex. So yeah, I think more collaboration in general is a good tool. (MV_11)

Community engagement and consultation were also frequently discussed when participants were asked about how negative outcomes of green gentrification could be limited, but it was not always clear what outcomes they believed this would mitigate. This suggestion appeared to be, at least in part, because it was one of the only tools that fell within the scope of their work. However, many noted the difficulties and limitations of meaningful community engagement, including a lack of clear agenda-setting and a current focus on education versus engagement.

I think the city can get a lot better in how we do consultation. I think we can get a lot better at setting agendas in terms of ‘here's where we're at, here's what is up for debate and here's what is not up for debate. Like we're not here to debate human dignity and certain things’. But we can, I think, get a lot better at setting the agenda as opposed to going, ‘we're just ticking a box, we have to do a consultation, let's send three planners to sacrifice and they'll just stand there for an hour and get yelled at and nothing constructive happens’. (GTA_13)

There's still definitely, in both organizations I worked at, more kind of the idea of what needs to happen is just education and not engagement or power-shifting or anything like that. (GTA_4)

Some were skeptical that community engagement would even limit negative impacts because it may be used by some stakeholders to further their own financial interests or because community-driven greening could still be co-opted into gentrification processes. This further exemplifies the limited power interviewees felt they had to mitigate green gentrification within their current positions and domains of work.

It's often my experience in working on land-use plans […] you may work with residents or community stakeholders who are ultimately looking to move, maybe looking at maximizing the profit and trying to get as much out of their own properties. So those are difficult discussions, right? … they may not have the community's long-term interest at heart, but they do speak on behalf of the current neighborhood. (MV_12)

I think there are some cool things being explored, like working with neighborhoods and lower-income neighborhoods to do participatory mapping of different opportunities and having the neighborhood sort of drive the type of work that happens in the area versus just the organization going in and being like, ‘this is what we want to do’. But I don't know, that could still result in gentrification. (GTA_9)

## Discussion

In this discussion, we explain how greening requirements imposed on new developments contribute to a *greening of the gentrification process*. Here greening is not a *cause* of gentrification (as defined in the concept of green gentrification) but frequently becomes linked to it via various political-economic factors and the deployment of a grey-green ideology of sustainability. We then highlight how this contributes to the reticence of many interviewees to ascribe greening as the sole driver of gentrification. We follow this with an overview of the power dynamics that exist between developers, policymakers, and urban greening planners/practitioners, and how this influences interviewees’ views on the intentionality of green gentrification and their ability to mitigate negative gentrification outcomes, primarily physical displacement. As we discuss at the end, despite a current focus on addressing existing inequities in their work, reliance on development to fund and accommodate urban greening appears antithetical to equitable greening outcomes and limits the utility of ‘green gentrification’ to inform equitable approaches to urban greening.

### Urban greening requirements and the ‘greening of gentrification’

Interviewees described a process in which political-economic factors – such as policy requirements for developer contributions to greening, desire to keep property taxes low, reduced provincial/federal funding, and high land values making cash-in-lieu greening contributions less desirable – result in a reliance on development to fund greening and lead to greening being placed alongside new developments (see also Reibel et al., (2021)) who found more park funding directed to gentrifying areas). The decline of purpose-built rentals and government funding for social and affordable housing (Taylor, 2019) has meant that most new developments are geared towards higher-income households – a point raised by many interviewees. Together, these findings raise the possibility that beyond instances of gentrification surrounding spectacular new greening initiatives (e.g. large parks, greenways, etc.), there may be a broader ‘greening of gentrification’ that is, at least in part, facilitated by city greening requirements levied on new developments. We refer to this as a broader ‘greening of gentrification’ because it suggests that beyond instances of greening *driving* gentrification, greening can become integrated into the gentrification process not as a *cause* of it but as a development requirement informed by the urban sustainability shift occurring within the neoliberal context (i.e. as a means of greening the city amidst reduced public funding). This overall greening of gentrification is likely rooted in the green-grey ideology of urban sustainability, in which sustainability is signified through pairing vegetative forms of greening with grey energy-efficient infrastructures (Wachsmuth and Angelo, 2018). Various requirements imposed by governments indicate an expectation for new developments to articulate their sustainability through the deployment of this green-grey ideology, contributing to this greening of gentrification. Recent research has indicated that greening is very commonly undertaken in gentrifying areas in Vancouver and Toronto (Quinton et al., 2023), suggesting that this sustainability shift in urban planning/policy results in widespread integration of greening into the gentrification process.

This notion of a broader greening of gentrification is not to say that greening is always the main driver of gentrification (see Anguelovski et al., 2022) or that all new greening will lead to gentrification (Anguelovski et al., 2017; Rigolon and Németh, 2020). Rather, political-economic factors frequently result in the co-location of urban vegetation alongside new developments targeted towards higher-income households. The result is an overall greening of the gentrification processes going on across these cities, which will perpetuate existing inequitable distributions of urban vegetation. The way in which urban greening becomes linked to gentrification via greening requirements on new developments clearly differs, for example, from the process of the Atlanta Beltline, in which the announcement of the greening investment induced speculation (Immergluck, 2009) or the NYC High Line, where prospective real-estate investment was used to rationalize greening the abandoned railway (Lang and Rothenberg, 2017). In such cases, greening is noticeably spurring on gentrification, whereas the imposition of greening requirements suggests development was going to happen regardless of prior greening. This may align with the notion of ‘gentrified greening’ (Sharifi et al., 2021) in cases where early gentrification attracts further development that generates funding for greening (Rigolon and Collins, 2022), but it may not always be the case that the area was already gentrifying. Greening requirements may ultimately attract higher-income households to move into the area, thus contributing to gentrification. However, it is also plausible that development targeted towards high-income households would be sufficient to gentrify the area without greening imposed by requirements, in which case greening could perhaps be viewed as an outcome of gentrification – or merely a correlating factor. This highlights the multiple, complex relationships that can exist between urban greening and gentrification, which extend beyond the green-gentrification notion of greening causing gentrification. In describing a broader greening of gentrification, we articulate how gentrification has internalized the urban sustainability shift and the implications that this has for the continuation of inequitable urban greening even if greening is not driving the gentrification process.

### The role of green and non-green factors in gentrification

Many interviewees highlighted that greening is not happening in isolation, as their greening work is often done as part of a larger (re)development process. This makes it difficult to determine how much of gentrification can be attributed to greening vs other non-green factors included in the development (e.g. transit, new housing, and other amenities). As such, many interviewees were sceptical that greening alone causes gentrification, which contrasts the centring of greening as a driver of gentrification seen in green gentrification literature. Limited research on this topic indicates variability in the role greening plays in gentrification relative to other factors (Anguelovski et al., 2022), and there has been little research into how greening intersects with other non-green factors during the gentrification process (Quinton et al., 2022). Related to this, some working in suburban municipalities with remaining greenfield areas noted that development often reduces the total amount of ‘greenness’ by converting open areas into developed land, leading them to question whether *green* gentrification is an accurate term.

While spatial analyses have highlighted that greening occurring closer to downtowns and previously gentrified areas are more likely to result in gentrification (Anguelovski et al., 2017; Pearsall and Eller, 2020; Rigolon and Németh, 2020), this nuance was not raised by interviewees. However, some could see certain types of greening being more readily associated with gentrification, which has been empirically demonstrated (Rigolon and Németh, 2020; Triguero-Mas et al., 2022). Specifically, they believed more distributed greening, such as street trees and green infrastructure, would be less associated with gentrification – although such associations have been found (Donovan et al., 2021; Shokry et al., 2020). It is unsurprising that interviewees were not familiar with these nuances, as most were not discussing green gentrification at work and had limited exposure to the concept.

In addition to raising the role of non-green interventions in gentrification, some interviewees believed greening is not causing gentrification but that gentrification outcomes of greened areas occur because, as discussed above, the funding and space for greening is obtained through development. Although there was an overall scepticism that greening is driving gentrification, it should be noted that there may have been some reticence to attribute gentrification to greening because many interviewees viewed urban greening as an overwhelmingly positive action. Further, there were concerns that green gentrification blames practitioners (who felt they had little control over green gentrification) and that this would make it more difficult for them to undertake greening work in the future if the concept of green gentrification were weaponized (e.g. by developers or politicians) to reduce greening efforts. Thus, some interviewees may have felt defensive about the notion of green gentrification and the implications it has for their work. Finally, Metro Vancouver and the GTA both have very high housing costs, which may have contributed to scepticism that greening was the problem in these regions.

### Power dynamics in greening and gentrification

Interviewees frequently described ways in which they had limited control over greening, including where it occurs and any negative social impacts resulting from it. Many highlighted that they can only ‘work with what they are given’ when it comes to locating sites for greening. Such locations are often dictated by where development is occurring, prices of alternative locations, and the difficulties municipalities have competing with developers to purchase land (as they cannot get into bidding wars or move forward quickly with purchases), indicating the degree to which development and high real-estate prices influence greening. However, this influence of development is at least in part due to municipalities imposing greening requirements on new developments, indicating that policymakers retain some control over how greening becomes connected to gentrification via development. This is particularly true in cases described by interviewees in which developers view greening requirements as detrimental to their profits rather than, as described in existing literature, something that can be leveraged to increase property values and sale prices (Garcia-Lamarca et al., 2019; Lang and Rothenberg, 2017; Quastel, 2009). Although these greening requirements are imposed on developers by cities, one interviewee noted the need to be careful about how much cities require from developers (e.g. in terms of greening or affordable housing provisions), due to concerns that such requirements might encourage developers to operate in other cities instead. This indicates that despite being subject to policy requirements, developers still have influence over said requirements.

Interviewees also noted a few ways in which market forces beyond their control were shaping gentrification outcomes. When defining green gentrification, most people noted the role of consumer preference, wherein higher-income households are attracted to move into the neighbourhood due to greening (e.g. Gould and Lewis, 2017), suggesting market demand for greening to be a large factor. Many also noted the dramatic unaffordability of current housing markets in these regions, which suggested an overall demand for housing regardless of greening. As a result, some believed gentrification would still be occurring in their city in the absence of greening.

Both private- and public-sector employees noted that although they can make recommendations on greening, it is often up to elected officials whether they get implemented. This, combined with the influence of development and broader market dynamics, informed interviewee perspectives that gentrification is an ‘unintended consequence’ of greening – at least, unintended by them. Some did note that developers and politicians are actively trying to gentrify neighbourhoods. Green gentrification is sometimes framed in literature as an unintended consequence of greening efforts (Kim and Wu, 2021; Pearsall, 2018) and other times as an intentional process of greening the growth machine (Checker, 2011; Loughran, 2014). Our findings suggest nuance in the intentionality of green gentrification, with those responsible for the implementation of greening viewing it as an unintended consequence due to their limited power over certain aspects of greening and housing.

However, municipal greening requirements levied on new developments, and the role of interviewees in planning parks and other greening alongside other developments in area or neighbourhood planning, indicates that planners and policymakers do play a role in linking greening to gentrification – regardless of the intentionality of such actions. Interestingly, unlike other cities, the City of Vancouver has an elected Park Board. As noted by one interviewee, those responsible for greening in Vancouver sometimes request the re-zoning of an area to fund greening projects through subsequent development. This suggests they have the power to play a more active role in green gentrification through advocating for (re)development in order to fund their greening initiatives. Many interviewees noted the need to collaborate with housing and other relevant departments/authorities to limit displacement, as building coalitions across departments/sectors could potentially increase their power However, it was clear that this was not something that interviewees were currently pursuing. Further, it was evident that despite efforts at community engagement, they still retain greater power over urban greening than residents.

### Can knowledge of ‘green gentrification’ support equitable greening?

Interviewees described their current work as focused on addressing existing green inequities, but many felt that the concept of green gentrification was not useful to support equitable greening. Some noted a tension between addressing existing inequities and limiting negative green gentrification outcomes because of the implication that greening should be reduced to do so – although whether this would even be effective is unclear both based on previous research (Chen, 2021; Rigolon and Németh, 2020) and our finding that greening is typically coupled with development, suggesting gentrification would be likely to occur even in the absence of greening. This tension between green equity and green gentrification arises out of the approach to greening described previously, wherein greening is often funded through development and located alongside it. While such an approach to greening may help achieve broad goals (e.g. city-wide canopy-cover or park targets), it makes it difficult to address existing green inequities because currently underserved areas either (i) get less greening due to a lack of development; or (ii) greening that does occur happens alongside development targeted towards higher-income households. Some interviewees noted there are other funding sources for greening projects, but the emphasis was on development-funded greening (i.e. ‘development paying for itself’). Our findings suggest that decoupling urban greening from development funding, or at least being more intentional about channelling such funding to non-gentrifying areas (see Reibel et al., 2021), could help mitigate negative green gentrification outcomes and provide greening to underserved areas – although it is important to note that greening efforts may still be co-opted by developers (or other actors) in such cases.

Interviewees who thought green gentrification was a useful term and concept sometimes had trouble explaining how it could be used, and most simply suggested it could raise awareness. Many interviewees were concerned about the negative connotations of ‘gentrification’. It is common for cities to avoid using ‘gentrification’, favouring instead words like revitalization, renewal, uplift, etc. (Slater, 2009). However, environmental/ecological/green gentrification was specifically coined to re-politicize what has been seen as the a/post/de-politicized process of urban greening (Angelo, 2020). Of course, our interviews were conducted with government employees, consultants, and those in large environmental organizations. Thus, we did not capture the opinions of activists, who may be more likely to use ‘green gentrification’ precisely because it is political.

The other major concern interviewees had about the term was that it did not come with clear solutions. For many, the term seemed to imply that greening (and by association, those working in urban greening) was to blame for gentrification and that mitigating green gentrification meant limiting greening. Only two of them referenced the ‘Just Green Enough’ hypothesis (Curran and Hamilton, 2012, 2018) explicitly, but many described a similar idea and disagreed with the premise (see also Rigolon et al., 2020). This aligns with points they raised about the role of non-green factors in gentrification and the role of development in funding and accommodating greening, which led many to believe greening was not driving gentrification, and thus, limiting greening would not address the issue. Several interviewees acknowledged having ‘green blinders’ that encourage them to focus solely on greening, which may also contribute to this opposition to limiting greening – but it is worth noting that numerous researchers and residents have also opposed it (Rigolon et al., 2020). Some interviewees had concerns that ‘green gentrification’ could be co-opted by developers or other interest groups looking to limit greening, thus making it more difficult to get urban greening done – something they highlighted as already being difficult given the many competing demands for limited space and budgets.

Although a policy toolkit has been devised to limit green gentrification (Oscilowicz et al., 2021), only one interviewee was aware of it, and as they noted, it requires a degree of interdepartmental collaboration not currently seen. The same trend has been found in the US, and community groups and residents are forming multisectoral coalitions to address siloed government approaches (Oscilowicz et al., 2023). As we discuss in the next section, the work of interviewees focuses on greening, as housing issues fell outside of their domain.

### Limiting negative gentrification outcomes and implications for future dynamics of greening and gentrification

Due to discomfort with the ‘Just Green Enough’ concept, when asked about how to limit negative impacts, interviewees focused more on urban greening processes and the need for affordable housing policies. Meaningful community engagement to centre residents in the planning of urban greening was proposed by many as a means to limit green gentrification. Although greening to reflect the needs/desires/values of existing residents may help mitigate displacement (Amorim Maia et al., 2020) and meaningful engagement can reduce experiences of exclusion (Mullenbach et al., 2019), the literature on urban agriculture indicates how such approaches can still be co-opted to serve gentrification (Alkon and Cadji, 2020; Sax et al., 2022a). Some interviewees were also sceptical about community engagement limiting displacement, and this could be in part because of the development-greening connection that exists and would unlikely be altered by community engagement regarding the greening alone. It is possible that community engagement was put forward because it was something those working in urban greening could do within the scope of their work, as most are not involved in housing or policy domains.

Many interviewees noted the need for policy to address housing affordability to limit physical displacement and hoped that other people were working on this. However, they highlighted potential trade-offs between greening and recent approaches to housing affordability, such as in Ontario's *More Homes Built Faster Act* (*2022*) which aims to speed up housing production and lower costs by reducing requirements for parkland provision and development charges that fund greening. Interviewees from the GTA were concerned about this change and its impacts on their ability to provide parkland in the future, contributing to their emphasis on the connection between greening and development. The City of Vancouver ran a year-long pilot relaxation of its tree protection by-law between 2021 and 2022 to speed up approval of residential building permits, affecting not only tree retention but also oversight of the requirement to plant replacement trees (Lazaruk, 2022). These recent actions indicate an emerging dimension of the tensions between housing affordability/development and greening in Canada, wherein housing affordability concerns are used to justify a reduction in greening that favours developers. This could have implications not only for urban greening in the future but also for green-gentrification dynamics in areas where housing affordability is reaching a crisis point. It could reverse the trend of the broader greening of gentrification we identified, and further perpetuate the notion that greening is more of a luxury item.

Green gentrification literature often highlights the need for social housing, renter protections, etc., to limit displacement surrounding new greening projects (e.g. Garcia-Lamarca et al., 2019; Oscilowicz et al., 2021; Rigolon and Christensen, 2019), but with a high proportion (around 30% in both regions as of the 2021 census) of residents paying > 30% of their household income^1^ towards housing costs, and long wait lists for subsidized housing in Toronto^2^ (84,583 households; City of Toronto, 2023), it is clear that the need for affordable housing far outstrips current supply. One of the interviewees also noted the need to carefully consider thresholds for affordable-housing requirements within inclusionary zoning policies out of concern that developers will take their business elsewhere. This highlights not only the power that developers have but the need for concerted multiscalar efforts to address the issue across Canada – the latter of which is made difficult by urban entrepreneurialism (Harvey, 1989). Further, housing represents only one facet of affordability: low-income households also need close proximity to affordable transit and services/amenities. All of this indicates the complexity of trying to improve affordability alongside urban greening and the difficulty of doing so within the current neoliberal capitalist system, in which urban greening is often funded through development.

### Limitations and future research

Due to our interest in those engaged in greening, our study focused primarily on urban greening planners/practitioners. Thus, it did not capture the perspectives of individuals working in other sectors that may influence the relationship between greening and gentrification (e.g. housing, social policy, transportation, etc.) or those identified by interviewees as having power to mitigate negative green gentrification outcomes (e.g. elected officials). As with any case-based qualitative analysis, our results will not generalize to every context.

Based on our study, future research should examine how recent efforts to address housing affordability are influencing equitable urban greening and green gentrification. As many interviewees were sceptical that greening itself was causing gentrification, future research should examine how greening intersects with other non-green factors in the gentrification process.

## Conclusions

Our research indicates that while urban greening planners/practitioners in Metro Vancouver and the GTA define green gentrification in alignment with current research, they do not necessarily see greening driving gentrification. In these cities, new developments often provide space and funding for greening through policy requirements or incentives that aim to have ‘development pay for itself’. This connection between greening and development contrasts the green-gentrification notion that greening *causes* gentrification and suggests there has been a broader *greening of the gentrification process* that extends beyond instances of greening driving gentrification. Our findings indicate that gentrification has internalized the urban sustainability shift, and as a result, greening is likely to be frequently found in gentrifying/gentrified areas even if it is not driving the process. This will ultimately result in upholding existing, and creating new, urban green inequities as vegetation is situated within high-income areas. The broader greening of gentrification we identified is likely to be affected by recent provincial policy changes impacting the GTA, which substantially reduce greening requirements in the name of housing affordability. This highlights an emerging dimension in the tensions between urban greening and housing affordability, which will have implications for future dynamics in the relationships between greening and gentrification.

Planners/practitioners currently focus on addressing existing green inequities rather than avoiding future gentrification and were resistant to the Just Green Enough approach. Instead, they suggested increased community engagement, ongoing collaboration with those working in housing and social policy, and the need for strong policy to support equity – however, most felt they did not have the power to limit negative green gentrification outcomes. This limited power also contributes to practitioners viewing green gentrification as an ‘unintended consequence’ of their work rather than an intentional sustainability fix or greening of the growth machine, as described in current literature. Opinions on the term ‘green gentrification’ and its usefulness to support green equity through their work were divided, with some believing it could raise awareness of the issue. Others disliked its negative connotations and lack of clear solutions, and the concept did not appear relevant to the development-driven greening seen in their cities. Overall, it is clear that current approaches to greening are ill-suited to address existing green inequities or to limit future gentrification. Cities – and higher levels of government – need to create and enforce affordability and greening policies and approaches that centre equity and de-centre uneven economic growth.

## Highlights

- New developments in Metro Vancouver and the GTA provide funding/space for urban greening, resulting in a broader ‘greening of gentrification’.
- Through the ‘greening of gentrification’, greening becomes tied to gentrification, upholding inequitable greening without necessarily causing it.
- The work of urban green planners/practitioners currently focuses on addressing existing green inequities rather than strategizing to limit future gentrification.
- They view green gentrification as an ‘unintended consequence’ of their work due in part to their lack of power over greening and housing.
- The concept of ‘green gentrification’ is seen as having limited utility to support more equitable urban greening.

## Notes

1. The Statistics Canada definition of housing affordability.

2. Vancouver does not keep a waitlist.

## References

[bibr1-25148486241236281] Aalbers MB (2016) The Financialization of Housing: A Political Economy Approach. Abingdon, Oxon: Routledge.

[bibr2-25148486241236281] Affolderbach J Schulz C (2017) Positioning Vancouver through urban sustainability strategies? The greenest city 2020 action plan. Journal of Cleaner Production 164: 678–685.

[bibr3-25148486241236281] Alkon AH Cadji J (2020) Sowing seeds of displacement: Gentrification and food justice in Oakland, CA. International Journal of Urban and Regional Research 44: 108–123.

[bibr4-25148486241236281] Amorim Maia AT Calcagni F Connolly JJT , et al. (2020) Hidden drivers of social injustice: Uncovering unequal cultural ecosystem services behind green gentrification. Environmental Science & Policy 112: 254–263.

[bibr5-25148486241236281] Angelo H (2020) How Green Became Good: Urbanized Nature and the Making of Cities and Citizens. Chicago: University of Chicago Press. Available at: http://www.bibliovault.org/BV.landing.epl?ISBN=9780226739182 (accessed 20 July 2022).

[bibr6-25148486241236281] Anguelovski I Connolly JJT Cole H , et al. (2022) Green gentrification in European and North American cities. Nature Communications 13(1): 3816. 10.1038/s41467-022-31572-1 PMC9250502 35780176

[bibr7-25148486241236281] Anguelovski I Connolly JJT Masip L , et al. (2017) Assessing green gentrification in historically disenfranchised neighborhoods: A longitudinal and spatial analysis of Barcelona. Urban Geography 39(3): 458–491.

[bibr8-25148486241236281] Anguelovski I Connolly JJT Pearsall H , et al. (2019) Why green “climate gentrification” threatens poor and vulnerable populations. Proceedings of the National Academy of Sciences 116(52): 26139–26143.

[bibr9-25148486241236281] Bingham AJ Witkowsky P (2022) Deductive and inductive approaches to qualitative data analysis. In: Vanover C Mihas P Saldaña J (eds) Analyzing and Interpreting Qualitative Research. Thousand Oaks: Sage Publications, Inc., pp. 133–148.

[bibr10-25148486241236281] Braun V Clarke V (2006) Using thematic analysis in psychology. Qualitative Research in Psychology 3(2): 77–101.

[bibr11-25148486241236281] Bunce S (2018) Sustainability Policy, Planning and Gentrification in Cities. Routledge equity, justice and the sustainable city series. Abingdon, Oxon ; New York, NY: Routledge.

[bibr12-25148486241236281] Checker M (2011) Wiped out by the ‘Greenwave’: Environmental gentrification and the paradoxical politics of urban sustainability. City and Society 23(2): 210–229.

[bibr13-25148486241236281] Chen Y (2021) Can smaller parks limit green gentrification? Insights from Hangzhou, China. Urban Forestry & Urban Greening 59: 127009.

[bibr14-25148486241236281] City of Toronto (2023) Social Housing Waiting List Reports. Toronto: City of Toronto. Available at: https://www.toronto.ca/city-government/data-research-maps/research-reports/housing-and-homelessness-research-and-reports/social-housing-waiting-list-reports/ (accessed 10 May 2023).

[bibr15-25148486241236281] Creswell J 2006) Qualitative Inquiry and Research Design: Choosing Among Five Approaches, 2nd Revised edition Thousand Oaks: Sage Publications.

[bibr16-25148486241236281] Curran W Hamilton T (2012) Just green enough: Contesting environmental gentrification in Greenpoint, Brooklyn. Local Environment 17(9): 1027–1042.

[bibr17-25148486241236281] Curran W Hamilton T 2018) Just Green Enough: Urban Development and Environmental Gentrification. New York: Routledge.

[bibr18-25148486241236281] Daoust M Hoffarth M Haines T (2021) Trends in the Canadian mortgage market: Before and during COVID-19. 17 February. Statistics Canada. Available at: https://www150.statcan.gc.ca/n1/pub/11-621-m/11-621-m2021001-eng.htm (accessed 28 June 2023).

[bibr19-25148486241236281] Dilworth R Stokes R (2013) Green growth machines, LEED ratings and value free development: The case of the Philadelphia property tax abatement. Journal of Urbanism: International Research on Placemaking and Urban Sustainability 6(1): 37–51.

[bibr20-25148486241236281] Donovan GH Prestemon JP Butry DT , et al. (2021) The politics of urban trees: Tree planting is associated with gentrification in Portland, Oregon. Forest Policy and Economics 124(August 2020): 102387. 10.1016/j.forpol.2020.102387 PMC8408830 34483719

[bibr21-25148486241236281] Dooling S (2009) Ecological gentrification: A research agenda exploring justice in the city. International Journal of Urban and Regional Research 33(3): 621–639.

[bibr22-25148486241236281] Garcia-Lamarca M Anguelovski I Cole H , et al. (2019) Urban green boosterism and city affordability: For whom is the ‘branded’ green city? Urban Studies 58(1): 90–112.

[bibr23-25148486241236281] Gerrish E Watkins SL (2018) The relationship between urban forests and income: A meta-analysis. Landscape and Urban Planning 170(November 2017): 293–308. 29249844 10.1016/j.landurbplan.2017.09.005 PMC5726445

[bibr24-25148486241236281] Goossens C Oosterlynck S Bradt L (2019) Livable streets? Green gentrification and the displacement of longtime residents in Ghent, Belgium. Urban Geography 41(4): 550–572.

[bibr25-25148486241236281] Gordon JC (2020) Solving puzzles in the Canadian housing market: Foreign ownership and de-coupling in Toronto and Vancouver. Housing Studies 37(7): 1250–1273.

[bibr26-25148486241236281] Gould KA Lewis TL (2017) Green Gentrification: Urban Sustainability and the Struggle for Environmental Justice. New York: Routledge.

[bibr27-25148486241236281] Grant A Millward AA Edge S , et al. (2022) Where is environmental justice? A review of US urban forest management plans. Urban Forestry & Urban Greening 77: 127737.

[bibr28-25148486241236281] Grube-Cavers A Patterson Z (2015) Urban rapid rail transit and gentrification in Canadian urban centres: A survival analysis approach. Urban Studies 52(1): 178–194.

[bibr29-25148486241236281] Gunder M Hillier J (2009) Planning in Ten Words or Less: A Lacanian Entanglement With Spatial Planning. Burlington, VT: Ashgate Publishing, Ltd.

[bibr30-25148486241236281] Harris B Rigolon A Fernandez M (2020) “To them, we’re just kids from the hood”: Citizen-based policing of youth of color, “white space,” and environmental gentrification. Cities 107(August): 102885.

[bibr31-25148486241236281] Harvey D (1989) From managerialism to entrepreneurialism: The transformation in urban governance in late capitalism. Geografiska Annaler. Series B, Human Geography 71(1): 3–17.

[bibr32-25148486241236281] Harvey D (2005) A Brief History of Neoliberalism. Oxford, UK: Oxford University Press, Incorporated. Available at: http://ebookcentral.proquest.com/lib/ubc/detail.action?docID=422896 (accessed 15 December 2022).

[bibr33-25148486241236281] Immergluck D (2009) Large redevelopment initiatives, housing values and gentrification: The case of the Atlanta Beltline. Urban Studies 46(8): 1723–1745.

[bibr34-25148486241236281] Jelks NO Jennings V Rigolon A (2021) Green gentrification and health : A scoping review. International Journal of Environmental Research and Public Health 18: 1–23. 10.3390/ijerph18030907 PMC7908481 33494268

[bibr35-25148486241236281] Keil R Graham J (1998) Reasserting nature: Constructing urban environments after Fordism. In: Braun B Castree N (eds) Remaking Reality: Nature at the Millennium. New York: Routledge, 98–124.

[bibr36-25148486241236281] Kim SK Wu L (2021) Do the characteristics of new green space contribute to gentrification? Urban Studies 59: 360–380.

[bibr37-25148486241236281] Konijnendijk CC Ricard RM Kenney A , et al. (2006) Defining urban forestry – A comparative perspective of North America and Europe. Urban Forestry & Urban Greening 4: 93–103.

[bibr38-25148486241236281] Lang S Rothenberg J (2017) Neoliberal urbanism, public space, and the greening of the growth machine: New York City’s High Line park. Environment and Planning A 49(8): 1743–1761.

[bibr39-25148486241236281] Lazaruk S (2022) At least 640 trees in Vancouver felled during year-long pilot project to relax tree protection. Vancouver Sun, 3 June. Available at: https://vancouversun.com/news/local-news/at-least-640-trees-in-vancouver-felled-during-year-long-pilot-project-to-relax-tree-protection (accessed 28 June 2023).

[bibr40-25148486241236281] Loughran K (2014) Parks for profit: The High Line, growth machines, and the uneven development of urban public spaces. City and Community 13(1): 49–68.

[bibr41-25148486241236281] Low S Taplin D Scheld S (2005) Rethinking Urban Parks: Public Space and Cultural Diversity. Austin: University of Texas Press. Available at: https://muse.jhu.edu/pub/15/monograph/book/2997 (accessed 15 May 2023).

[bibr42-25148486241236281] MacKinnon L High S , (2020) Deindustrialization. In: Kaltmeier O Tittor A Hawkins D et al. (eds) The Routledge Handbook to the Political Economy and Governance of the Americas. London: Routledge, 57–67. Available at: https://www.taylorfrancis.com/chapters/edit/10.4324/9781351138444-6/deindustrialization-lachlan-mackinnon-steven-high (accessed 15 December 2022).

[bibr43-25148486241236281] McCann E (2013) Policy boosterism, policy mobilities, and the extrospective city. Urban Geography 34(1): 5–29.

[bibr44-25148486241236281] Miller JT (2016) Is urban greening for everyone? Social inclusion and exclusion along the Gowanus Canal. Urban Forestry and Urban Greening 19: 285–294.

[bibr45-25148486241236281] Molotch H (1976) The city as a growth machine: Toward a political economy of place. American Journal of Sociology 82(2): 309–332.

[bibr46-25148486241236281] Moore AA (2016) Decentralized decision-making and urban planning: A case study of density for benefit agreements in Toronto and Vancouver. Canadian Public Administration 59(3): 425–447.

[bibr47-25148486241236281] Mullenbach LE Baker BL Benfield J , et al. (2019) Assessing the relationship between community engagement and perceived ownership of an urban park in Philadelphia. Journal of Leisure Research 50(3): 201–219.

[bibr48-25148486241236281] Nesbitt L Meitner MJ Girling C , et al. (2019) Urban green equity on the ground: Practice-based models of urban green equity in three multicultural cities. Urban Forestry and Urban Greening 44(August): 126433.

[bibr49-25148486241236281] Nesbitt L Sax DL Quinton J , et al. (2023) Greening practitioners worry about green gentrification but many don’t address it in their work. Ecology and Society 28(4): 29.

[bibr50-25148486241236281] Oscilowicz E Anguelovski I García-Lamarca M , et al. (2023) Grassroots mobilization for a just, green urban future: Building community infrastructure against green gentrification and displacement. Journal of Urban Affairs: 1–34. 10.1080/07352166.2023.2180381 PMC11789711 39906343

[bibr51-25148486241236281] Oscilowicz E Lewartiwska E Levitch A , et al. (2021) Policy and planning tools for urban green justice: Fighting displacement and gentrification and improving accessibility and inclusiveness to green amenities. BCNUEJ. Available at: https://www.bcnuej.org/wp-content/uploads/2021/04/Toolkit-Urban-Green-Justice.pdf (accessed 11 May 2023).

[bibr52-25148486241236281] Parish J (2020) Re-wilding Parkdale? Environmental gentrification, settler colonialism, and the reconfiguration of nature in 21st century Toronto. Environment and Planning E: Nature and Space 3(1): 263–286.

[bibr53-25148486241236281] Patrick DJ (2014) The matter of displacement: a queer urban ecology of New York City’s High Line. Social and Cultural Geography 15(8): 920–941.

[bibr54-25148486241236281] Pearsall H (2018) New directions in urban environmental/green gentrification research. In: Lees L Phillips M (eds) Handbook of Gentrification Studies. Cheltenham: Edward Elgar Publisher, 329–345.

[bibr55-25148486241236281] Pearsall H Eller JK (2020) Locating the green space paradox: A study of gentrification and public green space accessibility in Philadelphia, Pennsylvania. Landscape and Urban Planning 195(February 2019): 103708.

[bibr56-25148486241236281] QSR International Pty Ltd (2020) Nvivo R1. Available at: https://www.qsrinternational.com/nvivo-qualitative-data-analysis-software/home.

[bibr57-25148486241236281] Quastel N (2009) Political ecologies of gentrification. Urban Geography 30(7): 694–725.

[bibr58-25148486241236281] Quinton J Nesbitt L Connolly JJ , et al. (2023) How common is greening in gentrifying areas? Urban Geography: 1–23.

[bibr59-25148486241236281] Quinton J Nesbitt L Czekajlo A (2022) Wealthy, educated, and …non-millennial? Variable patterns of distributional inequity in 31 Canadian cities. Landscape and Urban Planning 227: 104535.

[bibr60-25148486241236281] Quinton J Nesbitt L Sax DL (2022) How well do we know green gentrification? A systematic review of the methods. Progress in Human Geography 46(4): 960–987. 35971517 10.1177/03091325221104478 PMC9373194

[bibr61-25148486241236281] Reibel M Rigolon A Rocha A (2021) Follow the money: Do gentrifying and at-risk neighborhoods attract more park spending ? Journal of Urban Affairs 45(5): 1–19.

[bibr62-25148486241236281] Richards JC (2022) Coding, categorizing, and theming the data: A reflexive search for meaning. In: Vanover C Mihas P Saldaña J (eds) Analyzing and Interpreting Qualitative Research. Thousand Oaks: Sage Publications, Inc., pp. 149–167.

[bibr63-25148486241236281] Rigolon A (2016) A complex landscape of inequity in access to urban parks: A literature review. Landscape and Urban Planning 153: 160–169.

[bibr64-25148486241236281] Rigolon A Christensen J (2019) Greening without Gentrification: Learning from Parks-Related Anti-Displacement Strategies Nationwide. Los Angeles: UCLA Institute of the Environment & Sustainability. Available at: https://www.ioes.ucla.edu/wp-content/uploads/Greening-without-Gentrification-report-2019.pdf.

[bibr65-25148486241236281] Rigolon A Collins TW (2022) The green gentrification cycle. Urban Studies 60(4): 1–16.

[bibr66-25148486241236281] Rigolon A Keith SJ Harris B , et al. (2020) More than ‘Just Green Enough’: Helping park professionals achieve equitable greening and limit environmental gentrification. Journal of Park and Recreation Administration 38(3): 29–54.

[bibr67-25148486241236281] Rigolon A Németh J (2020) Green gentrification or ‘just green enough’: Do park location, size and function affect whether a place gentrifies or not? Urban Studies 57(2): 402–420.

[bibr68-25148486241236281] Rosol M Béal V Mössner S (2017) Greenest cities? The (post-)politics of new urban environmental regimes. Environment and Planning A: Economy and Space 49(8): 1710–1718.

[bibr69-25148486241236281] Saldaña J (2016) The Coding Manual for Qualitative Researchers. Thousand Oaks: Sage Publications, Inc.

[bibr70-25148486241236281] Sax DL Nesbitt L Hagerman S (2022a) Expelled from the garden? Understanding the dynamics of green gentrification in Vancouver, British Columbia. Environment and Planning E: Nature and Space 6(3): 2008–2028.

[bibr71-25148486241236281] Sax DL Nesbitt L Quinton J (2022b) Improvement, not displacement: A framework for urban green gentrification research and practice. Environmental Science & Policy 137: 373–383.

[bibr72-25148486241236281] Sharifi F Nygaard A Stone WM , et al. (2021) Green gentrification or gentrified greening: Metropolitan Melbourne. Land Use Policy 108(May): 105577.

[bibr73-25148486241236281] Shokry G Connolly JJ Anguelovski I (2020) Understanding climate gentrification and shifting landscapes of protection and vulnerability in green resilient Philadelphia. Urban Climate 31(March 2019): 100539.

[bibr74-25148486241236281] Slater T (2009) Missing Marcuse: On gentrification and displacement. City 13(2-3): 292–311.

[bibr75-25148486241236281] Statistics Canada (2021) 2021 Census Boundary files. Available at: https://www12.statcan.gc.ca/census-recensement/2021/geo/sip-pis/boundary-limites/index2021-eng.cfm?year=21 (accessed 15 May 2023).

[bibr76-25148486241236281] Statistics Canada (2022) To buy or to rent: The housing market continues to be reshaped by several factors as Canadians search for an affordable place to call home. 21 September. Statistics Canada. Available at: https://www150.statcan.gc.ca/n1/daily-quotidien/220921/dq220921b-eng.htm (accessed 28 June 2023).

[bibr77-25148486241236281] Taylor ZT (2019) Shaping the Metropolis: Institutions and Urbanization in the United States and Canada. Montreal & Kingston, CA: McGill-Queen’s University Press.

[bibr78-25148486241236281] Triguero-Mas M Anguelovski I Connolly JJT , et al. (2022) Exploring green gentrification in 28 global North cities: The role of urban parks and other types of greenspaces. Environmental Research Letters 17(10): 104035.

[bibr79-25148486241236281] Vining AR Boardman AE (2008) Public-private partnerships in Canada: Theory and evidence. Canadian Public Administration 51(1): 9–44.

[bibr80-25148486241236281] Wachsmuth D Angelo H (2018) Green and gray: New ideologies of nature in urban sustainability policy. Annals of the American Association of Geographers 108(4): 1038–1056.

[bibr81-25148486241236281] Walks A Hawes E Simone D (2021) Gentrification in large Canadian cities: Tenure, age, and exclusionary displacement 1991–2011. Urban Geography 42(5): 603–633.

[bibr82-25148486241236281] Wheeler S (2013) Planning for Sustainability: Creating Livable, Equitable and Ecological Communities, 2nd ed London: Routledge.

[bibr83-25148486241236281] While A Jonas AEG Gibbs D (2004) The environment and the entrepreneurial city: Searching for the urban ‘sustainability fix’ in Manchester and Leeds. International Journal of Urban and Regional Research 28(3): 549–569.

